# A view from the top: how South African women leaders shatter the glass ceiling

**DOI:** 10.3389/fsoc.2025.1601448

**Published:** 2025-10-08

**Authors:** Kholosa Mathetha, Nelesh Dhanpat

**Affiliations:** Department of Industrial Psychology and People Management, College of Business and Economics, University of Johannesburg, Johannesburg, South Africa

**Keywords:** South Africa, glass ceiling, women leadership, career advancement, success strategies, barriers

## Abstract

This study investigates how South African women leaders navigated the glass ceiling to reach senior leadership positions. We explored the barriers these leaders encountered and examined the strategies they used to overcome workplace obstacles. We conducted semi-structured interviews with 15 women holding senior leadership positions across various South African industries. We selected participants through purposive and snowball sampling and analyzed data through Interpretative Phenomenological Analysis (IPA) to identify recurring patterns and themes. The analysis revealed four critical themes. First, participants faced persistent organizational barriers, including inadequate career support mechanisms and institutional bias in promotion decisions. Second, successful advancement required three core strategies: mentorship, strategic networking, and continuous skills development. Third, participants developed leadership presence through what they termed “positive influential femininity,” with many women actively practicing female empowerment within their organizations. Fourth, work-life integration presented ongoing challenges, requiring robust support systems and flexible organizational policies. Despite reaching senior positions, participants encountered continuing barriers. Women reported hitting additional glass ceiling effects at C-suite level, whilst most faced persistent gender stereotypes that limited their progression to executive roles. However, many participants successfully mentored other women into leadership positions, creating advancement pathways for future female leaders. These findings provide evidence-based strategies for dismantling glass ceiling barriers and offer practical approaches for organizations seeking to accelerate women's leadership advancement in South Africa's evolving corporate landscape.

## Introduction

The role of women in leadership remains a significant topic in discussions on the evolving global leadership landscape (Pogrebna et al., 2024). Women's leadership is not only a matter of equity but a strategic imperative for societal and economic sustainability (Faugoo, 2024). Research demonstrates that gender-diverse leadership enhances organizational performance (Moreno-Gómez et al., 2018) and innovation (Hemmert et al., 2024). Despite progress, considerable challenges persist.

Women aspiring to leadership positions encounter various barriers, ranging from overt discrimination and prejudice to subtle structural inequities (Allen et al., 2021; Galsanjigmed and Sekiguchi, 2023). These challenges reinforce the glass ceiling, an invisible yet powerful barrier that limits women's advancement to top leadership roles (Zhang and Basha, 2023), as well as the glass cliff, where women are disproportionately appointed to leadership roles during crises, setting them up for failure (Morgenroth et al., 2020).

Although education and workforce participation have increased, women remain underrepresented in senior leadership across industries (Thelma and Ngulube, 2024). This underrepresentation stems not only from personal and professional choices but also from systemic biases and structural barriers that disproportionately affect women (Offermann and Foley, 2020; Pitsoe et al., 2023). Gender stereotypes and cultural norms continue to associate leadership with traditionally male characteristics, reinforcing perceptions that women are less capable leaders (Abdallah and Jibai, 2020).

The leadership experiences of South African women are particularly complex. Post-apartheid legal reforms, including the Employment Equity Act and gender equality provisions in the South African Constitution, have promoted gender parity in leadership (Eynon, 2017). These legislative frameworks have increased female representation in senior roles and fostered cultural shifts toward gender equality (Ramohai, 2019). However, despite these progressive policies, deeply ingrained organizational practices and societal norms continue to hinder women's leadership advancement, particularly in corporate settings (O'Brien et al., 2023).

The persistence of the glass ceiling in South African business highlights the need to examine the barriers that hinder women's leadership progression (Akinola and Naidoo, 2024). In recent years, organizations globally have witnessed increased women's workforce participation due to enhanced educational attainment, evolving societal attitudes regarding gender roles, and the implementation of affirmative action policies and governmental initiatives promoting women's empowerment (Abalkhail, 2017; Klasen, 2019; Taparia and Lenka, 2024). Although contemporary workplaces enable female entry into traditionally male-dominated occupations (Greed, 2022), leadership roles remain significantly male-dominated, with the World Economic Forum citing a global gender gap of 31.5% encompassing employment, labor market opportunities, and pay disparity (Avolio et al., 2024; World Economic Forum, 2022).

This disparity varies considerably across industries, with relatively higher female representation in healthcare (43%), food and beverage (30%), and manufacturing (29%), contrasted by markedly lower participation in agriculture (17%), aerospace and defense (18%), and automotive (19%) (Sil and Lenka, 2025). The South African context mirrors these global trends, with Stats SA”s (2024) women's labor report revealing male dominance in private sector management positions between 2014 and 2024, demonstrating the systemic nature of gender inequality in corporate leadership.

These barriers include external organizational constraints and internal factors such as confidence and self-perception (Sales et al., 2020). Women executives frequently cite limited networking and mentorship opportunities as significant impediments to career growth (Barkhuizen et al., 2022). Additionally, they often bear a disproportionate burden of balancing professional and family responsibilities, reducing their availability for advancement opportunities (Huang et al., 2020).

Whilst existing research has extensively documented women's underrepresentation in leadership globally (Goryunova and Madsen, 2024; Hanifah, 2021), there remains a significant gap in understanding the lived experiences of women who have successfully navigated to senior leadership positions within South Africa's unique post-apartheid socio-political context. Previous studies have predominantly focused on barriers (Akinola and Naidoo, 2024; Kiaye and Singh, 2013) rather than exploring the nuanced, experiential realities of women who have overcome these obstacles. This study addresses this gap by employing Interpretative Phenomenological Analysis (IPA) to examine the lived experiences of South African women in senior management, providing an in-depth exploration of their career advancement journeys. The post-apartheid South African context offers a distinctive setting where progressive legislative frameworks coexist with persistent cultural and organizational barriers, creating a complex landscape that warrants specific investigation. By focusing on successful women leaders' narratives and the strategies they employed, this research contributes novel insights into how systemic barriers can be navigated and overcome within this unique context.

This study explores career advancement barriers through the experiences of South African women in senior management across various sectors. By focusing on their narratives, it identifies the challenges they encounter and the strategies they employ to overcome them. Understanding these structural barriers is essential for addressing gender disparities in leadership (Gillard and Okonjo-Iweala, 2022). Moreover, this research informs organizational policies and legislative reforms, fostering more inclusive leadership pathways.

While significant progress has been made toward gender equality in leadership, substantial challenges remain. The glass ceiling persists, and women, particularly in South Africa, continue to face obstacles (Barkhuizen et al., 2022; Kobus-Olawale et al., 2021; Pitsoe et al., 2023). This study contributes to ongoing efforts to dismantle these barriers by providing a comprehensive understanding of women's leadership challenges and proposing actionable strategies to support their success. It examines the experiences of women who have successfully navigated barriers to senior leadership, assessing the impact of the glass ceiling, key strategies, work-life balance, and insights for aspiring women leaders.

### Theoretical background

The glass ceiling, a structural barrier to women's leadership advancement, remains a global challenge (Taparia and Lenka, 2022; Sunaryo et al., 2024). Despite progress in gender equality, women remain underrepresented in senior roles due to cultural norms, organizational biases, and historical legacies (Bohr and Granato, 2024; Clarke-Glover, 2024). In South Africa, these challenges are compounded by apartheid's lingering effects, creating additional obstacles, particularly for women of color (Makhutla et al., 2021; Kark et al., 2024). This study examines the factors contributing to the glass ceiling globally and within South Africa, comparing its effects across developing nations. It also explores strategies to overcome these barriers, providing insights into fostering gender equality in leadership (Vyas-Doorgapersad and Bangani, 2020; Torres et al., 2024).

### Theoretical perspectives on the glass ceiling

This study employs three complementary theoretical frameworks to understand women's leadership experiences: intersectionality theory, social role theory, and organizational bias theory. These theories were selected to provide a comprehensive lens for analyzing the complex, multi-layered barriers women face in career advancement, and they directly informed both the interview design and analytical approach. Various theories explain the persistence of the glass ceiling and its impact on women's career progression (Alobaid et al., 2020).

**Intersectionality theory** (Crenshaw, 1989) serves as the primary theoretical framework, recognizing that gender discrimination cannot be understood in isolation from other identity categories such as race, class, and age. In the South African context, this theory is particularly relevant as it illuminates how women experience compounded disadvantages that differ qualitatively from those faced by their (Clarke-Glover, 2024).

**Social Role Theory** (Eagly and Wood, 2012) provides understanding of how gendered socialization shapes leadership expectations and career trajectories. This theory explains how societal role assignments from childhood influence workplace behavior and career aspirations, creating internal and external barriers to women's advancement (Newman and Newman, 2020).

**Organizational Bias Theory** examines how seemingly neutral organizational structures and practices systematically disadvantage women through embedded masculine norms and practices (Heilman and Manzi, 2022). This framework directed attention to organizational policies, informal networks, and institutional practices that participants encountered, while providing analytical tools to understand how bias operates at structural levels.

### The glass ceiling in South Africa

South Africa's history of apartheid and entrenched patriarchal norms have perpetuated gender and racial workplace inequalities (Ndinda and Ndhlovu, 2022; Segalo, 2015). Apartheid-era segregation marginalized black South Africans and women, limiting their access to professional advancement (Aborisade and Ariyo, 2023; Souza, 2021). While policies such as the Employment Equity Act and Broad-Based Black Economic Empowerment (BBBEE) Act aim to redress these imbalances, women remain significantly underrepresented in senior leadership (Dreyer et al., 2021).

Despite comprising 45.5% of the economically active population, women hold only 26.5% of top management roles and 37.2% of senior management positions (Department of Employment and Labour, 2023; McKinsey and Company, 2024). These disparities highlight the need for targeted policies to enhance gender equity in leadership (Zhang and Basha, 2023; Srivastava and Nalawade, 2023). Comparing South Africa with other nations reveals common barriers such as gender stereotypes and work-life balance challenges. However, in South Africa, the intersection of race and gender exacerbates these issues (Carrim, 2021). While countries like the US and UK have stronger support networks and advocacy initiatives to address gender disparities, progress remains slow worldwide. The World Economic Forum's Global Gender Gap Report (2022) indicates that global gender parity is still 132 years away, with women holding only 28.2% of managerial positions (UN Women, 2023). Although women increasingly earn higher education qualifications and remain active in the workforce, leadership gaps persist (Chisholm-Burns et al., 2017; Lwamba et al., 2022).

## Materials methods

### Research method

This study employed Interpretative Phenomenological Analysis (IPA) within a qualitative framework to examine participants' lived experiences of the glass ceiling phenomenon in career advancement. IPA facilitates the exploration of shared meanings amongst individuals who have encountered similar phenomena, enabling participants to articulate their experiences authentically without external constraints (Creswell, 2012). The phenomenological tradition focuses on identifying commonalities in how participants experience and interpret specific phenomena, whilst simultaneously engaging in an interpretive process wherein researchers derive meaning from these lived experiences (Creswell, 2013). Crucially, IPA emphasizes detailed examination of human experience in ways that allow such experiences to be expressed through participants' own conceptual frameworks rather than through predetermined analytical categories (Smith et al., 2009). IPA was chosen because it prioritizes experiential meaning-making and interpretative depth, whereas inductive approaches primarily seek thematic patterns that align with predetermined research objectives rather than exploring how phenomena shape individual lived realities (Alase, 2017).

### Research philosophy

Research philosophy involves assumptions about how knowledge develops (Saunders et al., 2019). This study adopted interpretivism, which emphasizes subjective experiences (Bryman, 2016) and favors qualitative data (Collins and Hussey, 2014). It aligns with qualitative research's aim of exploring human experiences, social contexts, and cultural influences (Oranga and Matere, 2023). The interpretive approach enabled an understanding of the glass ceiling by examining women's perceptions and experiences in leadership roles.

To establish a conceptualized IPA research framework, coherent paradigmatic foundations are essential for understanding participants' lived experiences. This study integrates Guba's (1990) critical theory paradigm with Burnell and Morgan's (1979) interpretive paradigm. Critical theory establishes the phenomenological foundation, whilst the interpretive paradigm provides the analytical framework for understanding how the phenomenon impacts participants' lived experiences.

Ontology and epistemology guide qualitative research (Al-Ababneh, 2020). Ontology concerns the nature of reality, exploring existence and categorization (Ylönen and Aven, 2023; Norton, 2023). This study applied subjectivism, asserting that reality is shaped by personal experiences and emotions (Ryan, 2018). Within the IPA framework, this ontological positioning recognizes that participants' realities are constructed through their individual interpretations of career advancement experiences, particularly within South Africa's unique socio-political context. Epistemology examines how we acquire and evaluate knowledge (Saunders et al., 2019), influencing data collection and analysis. The epistemological stance acknowledges that knowledge emerges through participants' meaning-making processes, requiring the researcher to engage deeply with their interpretative frameworks to understand how they construct knowledge from their leadership experiences. Using these assumptions, interviews explored the impact of the glass ceiling on women in leadership.

This study employed interpretative phenomenological analysis (IPA), which examines how individuals interpret experiences rather than documenting events objectively (Nizza et al., 2021). IPA focuses on meaning-making and self-reflection (Rajasinghe, 2020; Burns et al., 2022). The phenomenological tradition underlying IPA requires researchers to position themselves within participants' experiential worlds, enabling authentic interpretation of how glass ceiling phenomena are experienced and understood by successful South African women leaders. It provided insight into women leaders' subjective interpretations, aligning with the study's objectives. This paradigmatic integration ensures that the research captures both the critical examination of structural barriers and the interpretive understanding of how participants navigate and make sense of these challenges within their career advancement journeys. The researcher aimed to capture participants' concerns and contextualize their meanings within leadership challenge.

#### Sampling strategy and sampling size

A non-probability sampling technique combined purposive and snowball sampling. Purposive sampling, which selects participants based on specific characteristics relevant to the research objectives, proved suitable. This approach enabled the intentional selection of women in senior leadership roles to gain insights into barriers, challenges, and coping mechanisms at high organizational levels. Snowball sampling enriched the data further by identifying additional participants through referrals, incorporating diverse perspectives. It expanded access to senior women leaders by relying on participant referrals, which is effective for reaching specialized populations (Naderifar et al., 2017). Inclusion criteria required participants to hold senior management or executive positions. The final sample comprised 15 women in senior leadership roles, aligning with (Braun and Clarke 2012) recommendation of a minimum of 15 participants for master's-level qualitative research. Data saturation occurred at the 13th interview, with no new themes emerging (Mwita, 2022). Three additional interviews confirmed saturation. Participant demographics are detailed in Table 1.

**Table 1:** Biographical profiles of women leaders.

**Participant**	**Demographics**	**Industry**	**Role/position**
WL 1	39, African 16 years' experience	Healthcare	General manager
WL 2	59, Colored 37 years' experience	Health	Head of wellbeing
WL 3	39, Colored 15 years' experience	Health insurance	Senior manager
WL 4	37, African 12 years' experience	Banking	Head product control governance and controls
WL 5	36, African 10 years' experience	Mining	Senior finance manager
WL 6	48, Colored 20 years' experience	Medical	Head of surgery
WL 7	39, White 15 years' experience	Banking	Head of finance
WL 8	47, African, 26 years' experience	NPO	Deputy director legal
WL 9	48, Indian 25 years' experience	Training	President of professional association
WL 10	52, White 34 years' experience	Freight	Sales tools manager
WL 11	43, African 21 years' experience	Government	Deputy director
WL 12	31, African 10 years' experience	FMCG	Quality compliance and performance manager
WL 13	47, White 27 years' experience	Logistics	Vice-president sales development
WL 14	65, Colored 45 years' experience	Higher education	University executive
WL 15	30, African 8 years' experience	Real estate	Group finance executive

The sample consists of 16 senior women leaders across various industries. The largest ethnic representation is African (7; 44%), followed by Colored (4; 25%), White (3; 19%), and Indian (1; 6%). Ages range from 30 to 65 years, with most participants in their 40s (6; 38%). Experience varies from 8 to 45 years, with 10–20 years (5; 31%) being the most common. The most represented industries are healthcare and finance-related sectors (6; 38%), followed by government, logistics, and education (5; 31%). Leadership roles include executives (3; 19%), directors (3; 19%), and various senior managerial positions.

### Data collection

This study used semi-structured interviews to gather in-depth data. These interviews facilitate exploratory research within a broad framework while allowing flexibility (Magaldi and Berler, 2020). Unlike structured interviews with predefined questions or unstructured ones with open-ended inquiries, semi-structured interviews balance structure and adaptability (Dawson, 2019). They provide greater freedom in data collection, enabling researchers to explore participants' experiences, viewpoints, and attitudes (Silverman, 2016). The researcher used an 11-question interview guide to examine the glass ceiling. Open-ended questions encouraged participants to reflect on their experiences, while the flexible format allowed follow-up questions for deeper insights. Sample questions included: can you describe your current role? And how do you perceive your role as a senior leader in your organization? All interviews were conducted online via Microsoft Teams. To ensure anonymity, interviewers did not record video, and cameras remained off. Interviews lasted approximately 60 to 75 min, allowing participants sufficient time to express their views. To ensure accurate data recording, the researcher used transcripts, written notes, and audio recordings. Participants provided informed consent, understanding the purpose and intended use of the data.

### Data analysis

The interpretative phenomenological analysis (IPA) methodology was employed to examine participants' lived experiences and meaning-making processes. This approach prioritizes participants' subjective interpretations while acknowledging the researcher's interpretative role in understanding these experiences. Data were analyzed using interpretative phenomenological analysis following Storey's (2011) four-stage analytical process. Stage one involved immersive reading of transcripts with preliminary margin notes documenting initial impressions and researcher reflexivity. Stage two identified and labeled emerging themes grounded in participant data whilst incorporating theories related to the study. Stage three examined thematic relationships, consolidating related themes into hierarchical superordinate and subordinate structures. Stage four produced comprehensive thematic tables with illustrative quotations and coherent analytical narratives.

In qualitative research, rigor is established through trustworthiness, which includes credibility, transferability, dependability, and confirmability (Schwandt et al., 2007). The researcher ensured credibility by using multiple data sources and interview methods. Transferability was supported by providing detailed descriptions of the research context, sample selection, and methodology. The researcher maintained dependability through a transparent audit trail, including raw data, field notes, and coding schemes. Confirmability was addressed by acknowledging the researcher's role in data collection and analysis.

### Ethical considerations

Ethics examines right and wrong, guiding researchers' conduct (Bos, 2020). Qualitative research, particularly in-depth interviews, requires careful ethical consideration (Arifin, 2018). This study followed the University of Johannesburg's ethical guidelines and received clearance (IPPM-2024-887(M)). Participants were informed of their right to withdraw at any time. Informed consent was ensured by providing clear information about their involvement. The researcher avoided biased language, maintained participants' privacy and anonymity, and adhered to APA referencing. Objectivity was upheld in both execution and presentation of findings.

### Findings

This study identified six key themes crucial to understanding women in leadership roles in South Africa: organizational culture and practices, leadership presence, mentorship and sponsorship, work-life integration, success strategies, and barriers. Each theme includes sub-themes that offer insights into women's leadership paths and the challenges of the glass ceiling (Table 2).

**Table 2:** Overview of themes and subthemes.

**Themes**	**Description**	**Subthemes**
T1: Organizational culture and practices	Organizational policies that define and impact employee conduct such as the systems, attitudes, beliefs, sets of values and rules.	•Lack of support for women's career advancement •Lack of career support mechanisms •Institutional bias
T2: Leadership presence	Possessing the attributes of a woman who can successfully lead, inspire and influence others in a variety of settings.	•Positive influential femininity •Female empowerment
T3: Role of mentorship and sponsorship	By giving access to opportunities, advice and advocacy, mentors and sponsors are essential in helping those from marginalized groups advance their careers. Sponsors open doors and speak out for others, while mentors outline the routes that are open.	•Career advocacy •Networking •Coaching and mentorship
T4: Work-life integration	Allowing employees to balance their professional and personal lives to accomplish both sets of duties in a balanced manner.	•Support systems •Balancing personal lifestyle and professional work
T5: Success strategies	Initiatives that help people improve both professionally and personally and reach their goals.	•Skills development •Supportive networks
T6: Barriers	Career advancement barriers that impede female employees from advancing in their jobs or rising to senior positions in the workplace.	•Glass ceiling •Lack of opportunities for C-suite positions •Stigma and negative stereotypes

## Discussion of results

### Theme 1: organizational culture and practices

The institutional architecture of contemporary organizations continues to perpetuate gendered hierarchies through seemingly neutral policies and practices that systematically disadvantage women's career progression.

### Lack of support for women's career advancement

The findings reveal that organizational structures, whilst appearing gender-neutral, are embedded with masculine assumptions about leadership and career progression that create invisible barriers for women. Rather than overt discrimination, participants encountered subtle yet pervasive institutional resistance to their advancement, manifesting through overlooked promotions and absence of systematic support for women's upward mobility.

“*The great myths that we must really deal with, is the fact that we think people who look like us, so people of color, men of color, are as interested in our upliftment as well... they just as guilty as the other counterparts in keeping black women in junior roles” (WL 15, 65, married, University Executive)*.

“*I think a lot of the industries I've worked in have always been predominantly male dominated... the issue around employment equity and affirmative action... sometimes the myth that comes with transformation, you know, white males don't have opportunities. That's not true” (WL 1, 39, married, general manager)*.

This finding exposes the complex intersectionality of exclusion, where gendered barriers transcend racial boundaries, revealing how patriarchal structures operate across demographic lines to maintain male dominance in leadership hierarchies (Cohen et al., 2020; Tabassum and Nayak, 2021). These barriers are intensified by societal expectations and gendered norms that exclude women from senior roles (Bishu and Headley, 2020; O'connell and McKinnon, 2021). (Taparia and Lenka 2022) highlight how the intersectionality of gender with race, class, and age further complicates women's advancement, calling for a nuanced approach to workplace equity and inclusion (Heilman and Manzi, 2022).

### Lack of career support mechanisms

The absence of formal reintegration policies following career breaks represents a critical organizational blind spot that disproportionately impacts women's career trajectories. This gap in support reveals how organizations systematically fail to account for gendered career patterns, particularly around childbearing and caregiving responsibilities.

“*Companies don't... on ramp to then ensure actively that you're honoring someone back into that so they don't necessarily think ‘oh now God if we've lost someone, possibly after the childbearing years, we've got to replace that person with a female” (WL 2, 59, married, senior manager)*.

This finding illuminates how organizational career models remain predicated on uninterrupted, linear progression, a masculine career archetype that penalizes women for biological and social realities. The metaphor of “off-ramping” and “on-ramping” reveals how organizations conceptualize women's careers as deviations from the norm rather than legitimate alternative pathways.

“*a lot of the learning and mentoring actually also happened after hours and outside of work” and “because I'm a female, and also because I'm a non-white female, I was not part of that circle, at all”*

The lack of informal mentoring and networking opportunities, more accessible to male employees (Harris, 2022), further restricts women's access to career-enhancing resources and support (Manyeke and Dhanpat, 2024; Pitsoe et al., 2023). The literature has long established that career breaks, often associated with childbearing and caregiving responsibilities, adversely impact women's career progression (Torres et al., 2024; White and Goriss-Hunter, 2021). The lack of informal mentoring and networking opportunities, more accessible to male employees (Harris, 2022), further restricts women's access to career-enhancing resources and support (Manyeke and Dhanpat, 2024; Pitsoe et al., 2023).

### Institutional bias

The persistence of male-dominated executive structures, despite numerical gender parity at lower levels, exposes the concentrated nature of gender exclusion at senior levels. This pattern suggests that organizational barriers intensify as women approach positions of genuine power and influence.

“*There's a lot of females in our organization... but again, it's about at what level, right? So it's also still very much a male dominated environment” (WL 1, 39, married, general manager)*.

“*In corporate, it's a man's world. You know, I think women have made some great strides in corporates, but if you really scratching many organizations, you still find that a lot of the executives, the lot of the EXCO positions, are men” (WL 2, 59, married, senior manager)*.

Male-dominated environments reported by participants suggest that organizational practices continue to reflect gendered assumptions about leadership, obstructing women's advancement to executive roles (O'Brien et al., 2023; Galsanjigmed and Sekiguchi, 2023). While organizational structures appear gender-neutral, they remain shaped by implicit male standards, reinforcing a persistent gender hierarchy (O'Connor, 2020).

### Theme 2: leadership presence

Women leaders strategically leverage feminine-coded leadership qualities to create transformative organizational cultures whilst simultaneously challenging traditional masculine leadership paradigms.

### Positive influential femininity

Rather than conforming to masculine leadership norms, participants demonstrated how feminine-associated qualities, emotional intelligence, relational acumen, and collaborative approaches, constitute legitimate and effective leadership styles. This represents a fundamental challenge to the masculine prototype of leadership that has historically dominated organizational discourse.

“*She really... challenge the status quo and was really the first woman on the EXCO and really taking all the men on, you know, really head on” (WL 8, 39, married, head of finance)*.

“*Reached out to see who can supervise me clinically and I met a lady called* ^***^and she stood up for me against some of the and ills in the society like the board members trying to take advantage and me trying to push clinical integrity” (WL 10, 48, single, president of professional association).

This finding reveals how women leaders who embrace rather than suppress feminine qualities create more inclusive and supportive work environments, fundamentally disrupting traditional power dynamics. The concept of positive influential femininity recognizes leadership qualities such as emotional intelligence, empathy, and relational acumen, which contribute to a supportive work environment (Griffiths et al., 2019; Lemoine and Blum, 2021). These attributes have been shown to foster trust and rapport, enhancing organizational commitment and job satisfaction (Galsanjigmed and Sekiguchi, 2023). Women leaders reported that exposure to positive female role models has supported their career advancement by providing equal opportunities, workplace support, and professional fulfillment (Giguère et al., 2023; Ladam et al., 2018). The findings reveal how feminine leadership traits strengthen team dynamics and promote career growth (Thompson, 2024).

### Female empowerment

The concept of “lifting as they rise” emerged as a distinctive leadership philosophy among participants. These finding challenges traditional masculine leadership models predicated on individual achievement and competition, revealing how women leaders conceptualize success as collective rather than individual advancement.

“*My team actually has more females than males... I'm enjoying that and I think my role... is to also be intentional... being active in the change that you want to see within the organization” (WL 4, 37, married, head product control governance and controls)*.

This participant's deliberate cultivation of female talent represents a form of institutional resistance, using positional power to disrupt traditional gender hierarchies within organizational structures.

“*I prefer to groom and develop. So, when I say my senior role, I look at people as a potential. Potential to learn, potential to grow all of that. And when I see staff members that are willing and uh able to grow into certain positions even if it means grooming them out of my company, I would. I would do that, and I've done that two or three times already. I see it as an opportunity to grow people.” (WL 10, 48, single, president of professional association)*.

This emphasis on empowerment aligns with recent research indicating that female leaders who promote mentorship and growth opportunities not only support individual career progression but also strengthen organizational outcomes by developing a pipeline of future leaders (Murphy et al., 2024; Thelma and Ngulube, 2024). Empowering female leadership fosters a workplace culture where women are encouraged to leverage their strengths and pursue ambitious career goals. By creating environments that support women's advancement (Du, 2024), female leaders actively address historical gender inequalities and contribute to sustainable career pathways for future generations (Thelma and Ngulube, 2024).

This theme demonstrates how women leaders navigate the double bind of leadership—being perceived as either too feminine (and therefore weak) or too masculine (and therefore inappropriate for women). The findings support Heilman and Eagly's (2008) research on the effectiveness of transformational leadership styles often associated with women.

### Theme 3: role of mentorship and sponsorship

The career trajectories of successful women leaders are fundamentally shaped by strategic relationships that provide both developmental support and institutional access, revealing mentorship and sponsorship as critical mechanisms for overcoming systemic barriers.

### Career advocacy

Career advocacy transcends traditional mentoring by involving active promotion of women's capabilities in spaces where they lack representation. This finding reveals how individual agency alone is insufficient for career advancement; women require institutional allies who can advocate for their capabilities within exclusive decision-making circles.

“*Support networks... build sponsors within the organization. My current job I got because I had three people that were really sponsoring me... They were promoting me when I wasn't there” (WL 14, 47, long-term life partner, vice-president)*.

This participant's experience illuminates the informal networks of power that operate parallel to formal recruitment processes. The necessity of multiple sponsors suggests that women require greater social capital than men to achieve equivalent career outcomes.

“*The manager there was male, but he had a lot of ladies and was very intentional about transformation and employing and getting black female professionals and professionals of color within his units. So, they flourished and moved up the ranks from AD and DD.” (WL 12, 43, married, deputy director)*

Intentional leadership commitment to transformation can create environments where women of color can flourish professionally. Participants shared that, early in their careers, senior colleagues advocated for them in rooms they were not present in, vouching for their work ethic and acting as references. Research emphasizes the significance of advocates—individuals in influential roles who elevate qualified women, creating a more inclusive workplace (Gierke et al., 2024; Thelma and Ngulube, 2024). Women who received advocacy were better equipped to navigate promotions and secure leadership roles, supported by advocates committed to breaking down institutional barriers (Schwartz et al., 2024).

### Networking

The participants' emphasis on networking reveals how career advancement at senior levels operates through relational rather than meritocratic mechanisms. This finding exposes the inadequacy of formal equal opportunity policies that fail to address informal networks of power and influence.

“*at a certain level it's no longer about you applying for a job, but it gets to a point where people recommend you for a job” (WL 9, 47, married, deputy legal director)*

“*I think networks are important, but I think there's also ways of getting to networks... I think it's a network within understanding the output of what you deliver” (WL 8, 39, married, head of finance)*

This insight reveals the hidden curriculum of leadership advancement the unwritten rules about how senior positions are actually filled. Women's historical exclusion from informal networks creates compounding disadvantages that formal policies fail to address.

“*build that trusted network and get to know people and yeah, don't just keep to yourself” (WL 11, 52, married, sales tools manager)*

Networking is seen as a strategy for career growth, with studies indicating that higher-level positions are often filled through personal referrals and professional networks rather than formal recruitment channels (Shaymardanov et al., 2023). Participants strongly believed in networking, as all mentioned its significance for career advancement, breaking gender barriers, and promoting gender equality. Effective networking, however, is not just about making connections; it involves creating a community for idea exchange, problem-solving, and mutual support (Woehler et al., 2021). Women leaders must examine gender disparities from entry to upper management and focus on empowering other women to realize their leadership potential and push boundaries in male-dominated environments (Cabrera-Muffly, 2021).

### Coaching and mentorship

The distinction participants made between mentors (providing industry-specific guidance) and coaches (offering broader leadership development) reveals the multifaceted support required for women to navigate complex organizational hierarchies. This finding suggests that women require more comprehensive developmental support than their male counterparts due to additional barriers they face

“*I mean, that's what women are good at, knowing their purpose. But articulating that purpose, sometimes I think it becomes problematic. So, be quite deliberate about that. I think get yourself a coach and a mentor within the organization and preferably if you can an executive to spend some time with you, that's quite good.”* (WL 2, 59, married, senior management)

“*Also having mentors within the same sphere has helped a lot because they have insight. You know in Xhosa they say ‘indlela ibuzwa kwabaphambili,' so we learn from those who have journeyed before you.”* (WL 12, 43, married, deputy director)

“*I've got an executive coach. I've been with the coach now, probably for 4 years, so ever since I entered the senior management level, my executive coach has just been incredible and of course my mentor and my coach, two separate people, because I go to them for two separate umm, you know, avenues of advice.”* (WL 3, 39, single, senior management)

Mentoring was seen as crucial for moving between management levels, with a key barrier identified as the lack of mentoring opportunities (Seehusen et al., 2021). Recent literature suggests that coaching is vital for senior leaders, offering structured support to enhance leadership and interpersonal skills (Cidral et al., 2023; Subramanian et al., 2024). This study supports the notion that women are less likely than men to receive mentorship, with developmental connections being crucial for their career advancement (Shen et al., 2022).

### Theme 4: work-life integration

Women leaders reconceptualise work-life balance as strategic integration, challenging organizational assumptions about career commitment whilst maintaining professional excellence and personal wellbeing.

### Support systems

The diversity of support systems participants relied upon spanning religious, familial, collegial, and professional networks and reveals how women leaders create comprehensive scaffolding to sustain their careers. This finding challenges the individualistic assumption underlying traditional career models, demonstrating how successful women's leadership requires collective support structures.

“*Prayer and having a support system outside of work provided encouragement and focus” (WL 6, 48, married, head of surgery)*

“*You need the support of your friends, colleagues, industry, and family” (WL 9, 47, married, deputy legal director)*

“*Having a trusted colleague whom you admire for her achievements and leadership style is crucial” (WL 11, 52, married, tools sales manager)*

The diversity of support systems participants relied upon—spanning religious, familial, collegial, and professional networks—reveals how women leaders create comprehensive scaffolding to sustain their careers. This finding challenges the individualistic assumption underlying traditional career models, demonstrating how successful women leadership requires collective support structures.

Support systems are essential for women's career advancement and wellbeing, reinforcing findings on the positive impact of social and structural support in leadership (Ali et al., 2024; Heath and Weber, 2020). This sub-theme highlights the diverse support networks female leaders rely on throughout their careers. Effective support systems extend beyond the workplace, encompassing family, colleagues, organizational leaders, and spiritual affiliations, which serve as key sources of resilience (Guha and Rajesh Kadam, 2024; Taparia and Lenka, 2024). Participants emphasized the role of these networks in fostering courage, empowerment, and perseverance. Support systems not only facilitated career progression but also helped women navigate challenges, reinforcing the need for holistic support structures in leadership development.

### Balancing personal lifestyle and professional work

Participants' approaches to work-life integration reveal strategic resistance to organizational cultures that demand total availability. Their emphasis on boundaries and prioritization challenges the “ideal worker” norm that assumes unlimited commitment to professional demands.

“*Work-life integration is ideal. I prioritize essentialism—focusing on what matters most to me first, then addressing others' needs” (WL 10, 48, single, president of professional association)*.

“*The work environment and organizational culture are critical for work-life balance. Many women struggle with career and family demands, but where flexibility exists, balance becomes possible. Setting boundaries and managing time effectively helps navigate these challenges.”* (WL 16, 30, married, group finance executive).

Achieving meaningful work-life integration remains a significant challenge for women in leadership, as they navigate complex pressures requiring strategic adjustments (Chisholm-Burns et al., 2017). Findings indicate that women leaders recognize the necessity of balancing career, family, and personal growth, often requiring trade-offs. Some took career breaks to recalibrate, while others actively managed their dual responsibilities. Participants acknowledged the distinction between professional and personal domains, advocating for their effective management rather than conflation.

Organizational support plays a crucial role in enabling employees to balance these demands. Companies that foster work-life integration benefit from higher job satisfaction, employee retention, and productivity (Ali et al., 2024; Shahani et al., 2021). These findings reinforce the importance of integrating work and personal life while underscoring the organizational responsibility to implement policies that empower women to succeed in both spheres.

### Theme 5: success strategies

Women leaders develop sophisticated strategic approaches that combine skill development, behavioral adaptation, value-driven leadership, and network cultivation to navigate gendered organizational barriers whilst maintaining authentic leadership styles.

### Skills development

The emphasis on continuous learning and skill development reveals how women leaders compensate for institutional barriers through superior preparation and competence. This finding suggests that women require higher levels of qualification and preparation than men to achieve equivalent recognition and advancement.

“*Obviously, it requires a combination of life, you know, personal development, strategic career moves, then environment that you have around you. Self-belief and confidence are things that you can't buy in a store. You have to give it to yourself” (WL 16, 30, married, group finance executive)*.

“*I think it's learning, doing research for one is one of my strategies. Ensuring that I'm prepared for everything and also showing a very big willingness to take on challenges and bigger responsibilities” (WL 12, 43, married, deputy director of department)*.

Findings indicate that women in leadership require diverse skill sets, though their categorization varied. Skills development and relevance were closely linked, as participants emphasized the necessity of maintaining up-to-date knowledge within their fields. In several instances, these aspects were treated as distinct yet complementary. Leaders highlighted the importance of staying current with industry standards, aligning with research indicating that continuous skills development enhances adaptability and leadership effectiveness in dynamic organizational environments (Shan and Wang, 2024; Shet, 2024). The classification of these skills reflects the multifaceted competencies essential for women leaders to sustain relevance and excel in their roles.

### Supportive networks

The importance of surrounding oneself with supportive networks was emphasized. Participants credited their professional success to strong peer networks, mentorship, and friendships with like-minded individuals.

“*I think also, you know, friends with the same values, same vision. I met a lot of ethical colleagues, and it's nice to brainstorm, network and also just do soundboard and test your thinking on certain things” (WL 3, 39, single, senior management)*.

“*I've got a close-knit group of friends, and we are all almost at the same level in terms of achievement. We are a support structure for each other… Sometimes we meet on the weekend over a glass of wine, and we can each share our challenges… We can also share our successes” (WL 9, 47, married, deputy director legal)*.

The findings indicate that the women leaders regarded meaningful interactions, both personal and professional, with individuals who aligned with their interests and passions as essential for career progression (Gardiner et al., 2024). These networks comprised individuals who served as sounding boards for ideas, provided motivation during setbacks, monitored progress, and facilitated sustained momentum. The support systems surrounding the women leaders played a crucial role in maintaining their focus and ambition. Research suggests that women leaders who cultivate meaningful professional and personal relationships develop greater confidence and resilience, qualities instrumental in navigating challenges and advancing in their careers (Clarke, 2011; Smith et al., 2012).

### Theme 6: barriers

Despite decades of equality legislation and organizational diversity initiatives, women leaders continue to encounter systematic barriers that reveal the persistence of subtle yet powerful mechanisms of gender exclusion in senior leadership positions.

#### Glass ceiling

The glass ceiling's persistence demonstrates how invisible barriers operate to maintain male dominance in senior leadership, with intersectionality compounding these challenges for women of color.

“*People often say that glass ceiling is not there for black females... but sometimes there's only space for one black woman” (WL 9, 47, married, deputy legal director)*.

“I think my career trajectory through the various levels, from senior lecturer onward was very delayed and I spent a lot of time being a good organizational citizen. So, and you have that feeling that you're being needed and you sitting on selection committees and you're heading up all sorts of other little task teams. And then at some stage, it comes to you that actually, while you're doing this, your male colleagues are moving along quite swiftly up that academic trajectory, and everyone thinks you're a very nice person” (WL15, 65, married, University Executive).

The glass ceiling remains a formidable barrier to women's leadership advancement (Chisholm-Burns et al., 2017). Several participants reported being denied promotions and encountering systemic obstacles in progressing to senior roles due to gender bias. These findings align with extensive research on the glass ceiling (Cotter et al., 2001; Elacqua et al., 2009; Taparia and Lenka, 2024), a phenomenon in which women, despite possessing the necessary qualifications, face invisible barriers that hinder their ascent to top leadership positions (Cotter et al., 2001).

### Lack of opportunities for C-suite positions

Despite achieving leadership roles, many women leaders remain underrepresented at the highest executive levels due to systemic barriers that hinder access to top executive roles, including gender biases and inflexible organizational cultures.

“*We still have organizations where black people are still a minority in a country where black people are a majority... it's still white and male at the top” (WL 9, 47, married, deputy director legal)*.

“*But I can't get into a director position and that is because for most times I am female. So, for example, my director, she's female, she's been gunning for the CEO position. She cannot, you know, it's first your males and she knows it. There are male counterparts that have started even after her, they are new into the business, but they are looked at as candidates then she is” (WL5, 36, married, senior finance manager)*.

These findings align with research on gendered organizational cultures (Rutherford, 2014), which suggests that gender stereotypes and double standards create significant obstacles to women's leadership advancement (Mella, 2022; Thelma and Ngulube, 2024). In male-dominated leadership structures, implicit biases often prevent women from being perceived as suitable for C-suite roles (Montgomery, 2023; Whysall and Bruce, 2023).

### Stigma and negative stereotypes

The universality of stereotype experiences reveals how gendered assumptions about leadership capability continue to permeate organizational cultures, requiring women to constantly prove their competence whilst navigating patriarchal expectations about their “proper” roles.

“*Men still believe we belong in the kitchen... it's up to us to challenge that mentality that women can do this” (WL 13, 31, married, quality compliance manager)*.

“*There tends to be certain gender roles that are intrinsically within people that certain roles are more suited, or positions are more suited to a male than a female, and that kind of thinking. And obviously, we always be dealing with the patriarchy” (WL12, 43, married, deputy director of department)*.

This finding exposes the double bind women leaders face: they must simultaneously conform to feminine stereotypes to be accepted whilst demonstrating masculine-coded leadership qualities to be perceived as competent, a paradox that male leaders never encounter.

### Implications for practice

Based on the findings of the study, firstly, a significant role is played by organizational practices in shaping women's career trajectories to senior leadership roles. These observations are based on the fundamental need to create organizational cultures that respect diversity and inclusion. By destroying the implicit prejudices and institutional obstacles that have historically limited access to leadership roles for women, this research shows that cultural changes in organizations can provide actionable steps for their career advancement (Ackah et al., 2024; Kark et al., 2024).

Secondly, women leaders must navigate cultures that often lack structured career support, requiring them to proactively seek opportunities for growth and advocate for themselves. Moreover, emphasizing strategic initiatives such as mentoring and sponsorship programmes, the study underlines their great value as change agents. These initiatives help women's professional networks grow, provide the necessary advocacy required for career advancement, and close the ambition-opportunity gap (Bhattacharjee and Tiwari, 2024; Gillard and Okonjo-Iweala, 2022).

Thirdly, the need for effective work-life integration reflects the ongoing challenge of balancing professional and personal responsibilities. The findings showed that support systems and flexible policies are essential for women leaders to thrive. Furthermore, the study strongly supported the use of flexible work schedules as a key tactic to improve work-life balance and support the participation of women in leadership roles. Women leaders should also advocate for policies that facilitate better work-life integration. Organizations that implement these policies show their dedication to helping each person grow personally and also project themselves as forward-looking entities in a workforce that is fast changing (Sales et al., 2020; Mistry et al., 2024).

By concentrating on these pragmatic consequences, the study offered a complete road map for companies seeking to increase gender parity and raise their leadership ranks using a tapestry of many voices and points of view.

## Recommendations

Our findings have key practical implications. Firstly, we recommend the enhancing of organizational policies as they strengthen organizational diversity and inclusion policies by including active support mechanisms clearly meant to help women if they want to create an inclusive atmosphere that advances gender equality (Feeney and Stritch, 2019). Secondly, we propose programmes for development in leadership, as closing the current disparity between talent and opportunity in organizations depends on funding leadership development initiatives especially targeted at women. Thirdly, establishing official mentoring and sponsorship programmes that are essential in offering the structural support needed to enable newly appointed female executives to reach more senior roles (Seehusen et al., 2021). Finaly, we recommend the introduction of projects that are aimed at work-life balance to reduce the demands placed on women juggling personal and professional obligations depends mostly on using flexible work schedules and supporting societal changes that normalize work-life balance ( Huang et al., 2020; Mistry et al., 2024).

Future studies should explore the complex aspects of the glass ceiling phenomenon in other spheres and geographical areas throughout South Africa. Comprehensive knowledge of the professional paths taken by women leaders over lengthy times depends on longitudinal research. Unlike cross-sectional studies capturing a single moment in time, longitudinal research can expose the changing difficulties, and adaptive methods women use across their careers (Bhattacharjee and Tiwari, 2024; Santamaría et al., 2022). Additionally, future studies should pay more attention to intersectionality to investigate how other identity elements, including race, socioeconomic background, and other dimensions, along with gender interact to create leadership possibilities.

## Limitations

The study's limitations include the sample, which comprised women leaders from Gauteng and the Western Cape, thus representing only a specific segment (urban formal sectors) of South Africa's women leaders. As a result, the findings may not fully capture the experiences of women in other provinces of the country. Future research should expand the geographical scope to include women leaders from additional provinces in South Africa to offer a more comprehensive understanding of their challenges and experiences. The absence of women who did not reach leadership positions limits the findings' generalisability, as the sample only captured successful leadership trajectories despite all participants facing barriers, and may not reflect the experiences of women who were unable to overcome such obstacles Furthermore, certain perspectives may be overrepresented in the interviews, as the participants were primarily women focused on advancing their careers. The use of IPA, whilst providing rich interpretative insights, limited the study's generalisability due to its focus on individual experiences rather than broader patterns across populations. Additionally, a social desirability bias may have influenced participants to provide responses they perceived as socially acceptable. As a result, they may have been inclined to present themselves in a more favorable light and may have been hesitant to discuss negative experiences that could reflect unfavorably upon them.

## Conclusion

The aim of this study generally emphasized the need for change toward more inclusive policies that acknowledge and use the special difficulties and capabilities of women in leadership. Such studies are essential in ensuring that policy and practice develop together to meet the requirements of an increasingly varied workforce as we continue to see the speed of change in both local and worldwide settings. The road to tearing down the glass ceiling is long and calls for coordinated work in all spheres of society. Nonetheless, by matching study with concrete action, there is great potential to create workplaces that not only are fair but also enhanced by complete involvement of women leaders (Mistry et al., 2024; Poma and Pistoresi, 2024). By doing this, we get closer to a society in which all kinds of leadership capabilities are acknowledged and encouraged instead of being limited by gender or color.

## Data Availability

The raw data supporting the conclusions of this article will be made available by the authors upon reasonable request, without undue reservation.
